# Clinical application value of pre‐pregnancy carrier screening in Chinese Han childbearing population

**DOI:** 10.1002/mgg3.2425

**Published:** 2024-04-01

**Authors:** Li Tan, Yuefan Qi, Peijuan Zhao, LanLan Cheng, Guo Yu, Dongmei Zhao, Yu Xia Song, Yun Gai Xiang

**Affiliations:** ^1^ Department of Reproductive Medical Center The Second Affiliated Hospital of Zhengzhou University Zhengzhou China; ^2^ Department of Medical Imaging The Second Affiliated Hospital of Zhengzhou University Zhengzhou China; ^3^ Department of Reproductive Genetics Pingdingshan Maternal and Child Health Hospital Pingdingshan China

**Keywords:** monogenic/single gene disease, pre‐conception expanded carrier screening (PECS), preimplantation genetic testing of monogenic disease embryo, prenatal diagnosis

## Abstract

**Background:**

To explore the clinical application value of pre‐conception expanded carrier screening (PECS) in the Chinese Han ethnicity population of childbearing age.

**Methods:**

The results of genetic testing of infertile parents who underwent PECS in the Reproductive Medicine Center of the Second Affiliated Hospital of Zhengzhou University, China, from September 2019 to December 2021, were retrospectively analyzed. The carrier rate of single gene disease, the detection rate of high‐risk parents, and the clinical outcome of high‐risk parents were statistically analyzed.

**Results:**

A total of 1372 Chinese Han ethnicity patients underwent PECS, among which 458 patients underwent the extended 108‐gene test, their overall carrier rate was 31.7%, and the detection rate of high‐risk parents was 0.3%. The highest carrier rates were *SLC22A* (2.4%), *ATP7B* (2.4%), *MMACHC* (2.2%), *PAH* (1.8%), *GALC* (1.8%), *MLC1* (1.3%), *UNC13D* (1.1%), *CAPN3* (1.1%), and *PKHD1* (1.1%). There were 488 women with fragile X syndrome—*FMR1* gene detection, and 6 patients (1.2%) had *FMR1* gene mutation. A total of 426 patients were screened for spinal muscular atrophy—*SMN1*, and the carrier rate was 3.5%, and the detection rate of parents' co‐carrier was 0.5%.

**Conclusion:**

Monogenic recessive hereditary diseases had a high carrier rate in the population. Pre‐pregnancy screening could provide good prenatal and postnatal care guidance for patients and preimplantation genetic testing for monogenic/single gene disorders (PGT‐M) and prenatal diagnosis could provide more precise reproductive choices for high‐risk parents.

## Graphical abstract

The 108 extended genes testing and spinal muscular atrophy SMN1 gene carrier screening process. The sequential screening mode for both the husband and wife, that is, blood was first drawn from the wife for examination, and then the husband was tested for the screening items containing positive pathogenic genes when they were found to be carriers.

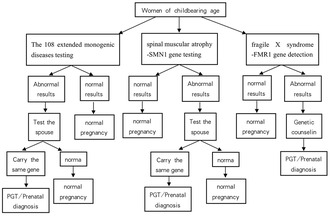

## 1 INTRODUCTION

Hereditary monogenic disease is one of the important causes of neonatal birth defects. According to epidemiological statistics, monogenic disease affects 1% of the live births and accounts for 10% of the infant mortality rate and 20% of the pediatric hospitalization rate (Kumar et al., 2001), which has a severe effect on the quality of the birth population and family happiness. Generally, heterozygous carriers of autosomal recessive hereditary diseases have no clinical symptoms. If both male partner and female partner carry the pathogenic gene of the same autosomal recessive hereditary disease, or women carry the pathogenic variation of X‐linked, sex‐linked diseases, the risk of bearing offspring with monogenic diseases increases. Therefore, pre‐conception expanded carrier screening (PECS) is a safe and effective primary prevention measure to provide more precise reproductive choices for high‐risk parents. The important purpose of PECS is to find individuals who carry pathogenic mutations (Zhao et al., 2021). Ideally, parents should undergo relevant gene testing before planning pregnancy to identify the carrier status of the individual or both the male partner and female partner. Doctors can provide detailed genetic counseling and reproductive strategies to avoid reproductive risk, and in case of high‐risk parents, pre‐embryo implantation genetic diagnosis or prenatal diagnosis can be selected to make informed and autonomous decisions regarding their reproductive choices (Wienke et al., 2014).

Initial carrier screening focused on certain high‐risk conditions specific to a particular race or family. With the increasing awareness of monogenic diseases and the development of genome sequencing technology, more and more pathogenic genes of monogenic hereditary diseases have been discovered, due to which the types of diseases screened by carriers continue to increase. Expanded carrier screening (ECS) was first provided in 2009 and is being used widely in clinical practice (Haque et al., 2016). Although ECS has a certain development basis, a unified standard and consensus have not yet been formed on the types of diseases included in the screening, how to explain the pathogenicity of the mutated gene, genetic counseling problems before and after testing, and the social impact of implementing ECS (Zhuang et al., 2019). This study was designed to screen the pre‐pregnancy genes of infertile patients who visited the Reproductive Medicine Center of the Second Affiliated Hospital of Zhengzhou University, to understand the carrier rate of 110 kinds of monogenic diseases among the childbearing population in Henan Province. This study also summarizes monogenic hereditary diseases with high carrier rate among the population and the corresponding pathogenic genes, in order to provide genetic counseling and valuable maternity guidance for high‐risk parents, and provide guidance and suggestions for good prenatal and postnatal care for all family members.

## 2 INFORMATION AND METHODS

### 2.1 Ethical compliance

This study was conducted with approval from the Ethics Committee of The Second Affiliated Hospital of Zhengzhou University (Approval number: 2022373). This study was conducted in accordance with the Declaration of Helsinki. Written informed consent was obtained from all participants.

### 2.2 Research participants

The research included infertile patients who visited the Reproductive Medicine Center of the Second Affiliated Hospital of Zhengzhou University from September 2019 to December 2021. The purpose and significance of pre‐pregnancy carrier screening was informed when looking for the cause or/and conducting pre‐operation examination of assisted reproductive technology; the informed consent form was signed and pre‐pregnancy carrier screening was conducted at the same time. All 1372 participants were of Chinese Han ethnicity. Among them, 380 female patients and 78 male patients received the expended 108 gene test. All female patients (488), were tested for fragile X syndrome—fragile X messenger ribonucleoprotein 1 (*FMR1*, OMIM#309550, NM_002024.6). A total of 415 female patients and 11 male patients were screened for spinal muscular atrophy—survival of motor neuron 1, telomeric (*SMN1*, OMIM#600354, NM_000344.4) gene carriers.

Pre‐pregnancy related gene testing was recommended for patients who met the following criteria: (1) patients who intended to receive fertility treatment using assisted reproductive technology; (2) infertile patients with unknown causes; (3) patients with history of adverse pregnancy and childbirth or recurrent abortion; (4) premature ovarian failure (POI); (5) both or one of the spouses has a hereditary disease or unexplained mental retardation; (6) patients who were willing to voluntarily undergo pre‐pregnancy gene testing. This study was approved by the Ethics Committee of the Second Affiliated Hospital of Zhengzhou University (Ethics Approval No.: 2022373).

### 2.3 Research methods

In this study, 110 monogenic diseases were selected as the screening items for pre‐pregnancy carriers, and each gene or related disease had the following characteristics: (1) severe clinical symptoms; (2) high carrier rate of pathogenic genes in screening population; (3) high reliability, specificity, and sensitivity of the detection method; (4) when abnormalities of these genes were detected, genetic counseling, PGT‐M, and prenatal diagnosis.

#### 2.3.1 Gene testing project

(1) Detection of 108 extended monogenic diseases: a total of 108 diseases with 129 detected genes. The categories of diseases screened included eight systems of musculoskeletal and connective tissue diseases, urinary system diseases, endocrine and metabolic diseases, nervous system diseases, blood and immune system diseases, and ophthalmology and otorhinolaryngology diseases; (2) Fragile X syndrome—*FMR1* gene detection; (3) spinal muscular atrophy—screening for carriers of the *SMN1* gene.

#### 2.3.2 Screening mode

**FIGURE 1:**
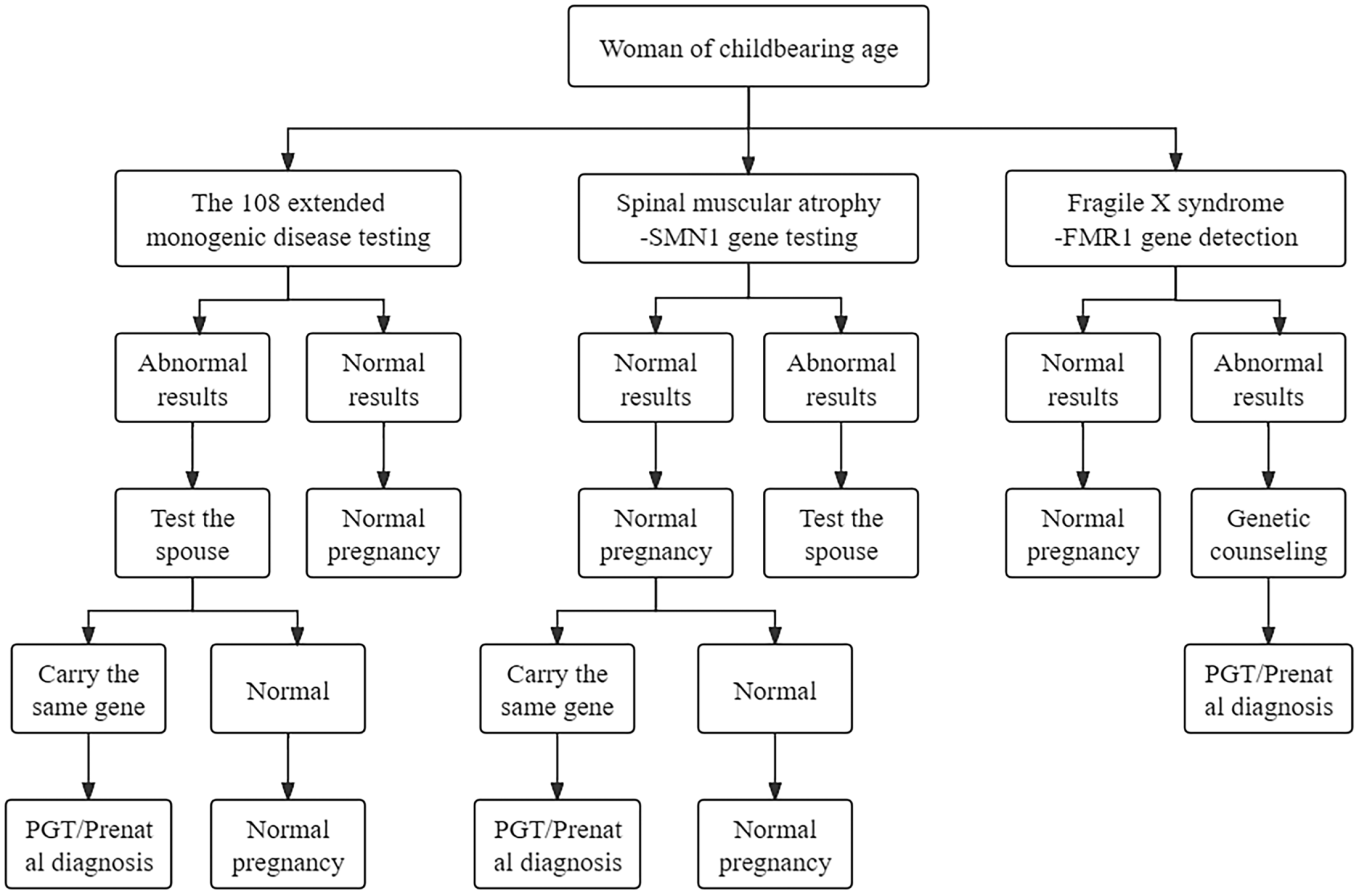
The 108 extended genes testing and spinal muscular atrophy *SMN*1 gene carrier screening process.

The sequential screening mode for both the male partner and female partner, that is, blood was first drawn from the female partner for examination, and then the male partner was tested for the screening items containing positive pathogenic genes when they were found to be carriers. See Figure 1 for details.

### 2.4 Test method

Next‐generation sequencing (NGS)‐based capture sequencing technology was used to detect the gene mutation of 108 monogenic diseases. Three primers PCR and capillary electrophoresis were used to analyze the CGG repeat times in the promoter region of the *FMR1* gene, using equipment from Asuragen, USA. The exon deletion detection kit of surviving motor neuron 1 (*SMN1*) from Shanghai Medicore Technology Co. Ltd. was used to detect the copy number of *SMN1*.

NGS (Next‐Generation Sequencing) Implementation: Effective sequencing data were aligned to the reference genome (GRCh38) using BWA (Li & Durbin, 2009), resulting in initial alignment results in BAM format. The alignment results were sorted using SAMtools (Li et al., 2009), and duplicate reads were marked using Sambamba. Based on the initial alignment results (BAM file), SNP positions were identified using SAMtools, and their statistics and annotations were performed. ANNOVAR (Wang & Li, 2010) software was utilized for SNP annotation. CNV Detection: CoNIFER (Krumm et al., 2012) software was employed to detect CNVs by analyzing the depth distribution of reads from the samples on a reference genome. Copy number variations were annotated using ANNOVAR software.

#### 2.4.1 Expanded 108 gene detection step

Sample DNA was extracted in the laboratory, the genomic DNA library was constructed, and then the exon DNA library was amplified by PCR. In the constructed DNA small fragment library, two ends of each insertion fragment were sequenced, and 150 bp was measured at each end. The original sequencing data were converted into an original sequencing sequence through base recognition analysis on an original image data file obtained using an Illumina sequencing platform, and finally the quality of the data was analyzed.

#### 2.4.2 Spinal muscular atrophy‐*SMN1* gene detection steps

Collect 2 mL of EDTA anticoagulated whole blood from all participants and extract genomic DNA using the Ex‐DNA Whole Blood Genomic DNA Extraction Kit (version 3.0). Amplify the SMN1 gene using specific primer sequences provided by Suzhou Tianlong Biotechnology Co., Ltd. through the SMN1 Gene Detection Kit. Assess the status of the SMN1 gene by observing the gel electrophoresis image. Subsequently, use computer software to calculate the ΔCT value by analyzing the fluorescence signals of SMN1 exons 7/8 and the reference gene CFTR from different samples. Based on the ΔCT values of each sample, calculate the calculated copy number (absolute value) for each sample and obtain the predicted copy number (integer value) based on the calculated copy number.

#### 2.4.3 *FMR1* gene detection steps

DNA Extraction: collect 2 mL of EDTA anticoagulated whole blood from all participants and extract DNA using the Magen Whole Blood DNA Extraction Kit from the United States. Follow the instructions provided with the extraction kit carefully.

Detection of FMR1 Gene CGG Repeat Sequences: After extracting genomic DNA, perform PCR amplification of the FMR1 gene using the FMR1 Gene Amplide Kit (Asuragen, USA) employing X detection technology and fluorescence PCR technology. Capillary electrophoresis is conducted using the 3730 DNA Analyzer capillary electrophoresis instrument provided by ABI (USA). The resulting amplification products are analyzed using GeneMapper 4.0 software, also provided by ABI (USA), to determine the number of CGG repeat sequences in the FMR1 gene. In the normal population, the CGG amplification fragment length ranges from 245 to 360 bp. The length of the intermediate CGG amplification fragment ranges from 363 to 389 bp. The length of the premutation CGG amplification fragment ranges from 392 to 820 bp. The length of the full mutation CGG amplification fragment is greater than 820 bp. The reaction system includes three primers (FMR1 forward primer, reverse primer, and CGG forward primer).

### 2.5 Observation indicators

The overall detection rate of relevant pathogenic gene variation of hereditary monogenic disease, the detection rate of high‐risk parents, and the clinical outcome of high‐risk parents. Evaluate pathogenic gene variation based on ACMG classification system (Richards et al., 2015).

## 3 RESULTS

### 3.1 Detection results of 108 expanded genes

#### 3.1.1 Sample size for detection of 108 expanded genes

In this study, among the 380 female patients who were tested for the expanded 108 genes, 121 patients were found to be carriers of monogenic hereditary disease, and a total of 140 pathogenic mutation genes were detected. The carrier rate of pathogenic genes for the target disease was 31.8% (121/380). Among the male partners of 121 carriers, 78 patients were willing to undergo related gene testing, and 24 patients were found to be carriers of monogenic disease. A total of 26 pathogenic mutation genes were detected, and the carrier rate of pathogenic genes for target disease was 30.8% (24/78). A total of 1 high‐risk parent with the same pathogenic gene AGL (glycogen storage disease type III) was detected among the 78 parents, with the detection rate of 0.3%.

#### 3.1.2 Carrier status of pathogenic genes of 108 target diseases

A total of 458 patients were tested for the expanded 108 genes. A total of 145 patients (31.7%) of target disease carriers were detected, including 129 patients (28.2%) carrying 1 pathogenic gene, 12 patients (2.6%) carrying 2 pathogenic genes, 3 patients (0.7%) carrying 3 pathogenic genes, and 1 patient (0.2%) carrying 4 pathogenic genes. Nine of the 129 pathogenic genes were carried in more than 1%, and the rest were less than 1%. The carrier status of the first nine pathogenic genes detected is shown in Table 1.

**TABLE 1:** The first nine pathogenic genes were detected by the extended 108 gene detection [*n* (%)].

Gene	Related diseases	OMIM No.	HUGO HGNC‐approved gene symbols	GenBank reference sequence and version number for the gene(s) studied^a^	Carrier status
*SLC22A5*	Systemic primary carnitine deficiency (CDSP)	603377	*SLC22A5*	NM_003060.4	11 (2.4)
*ATP7B*	Lenticular degeneration	606882	*ATP7B*	NM_000053.4	11 (2.4)
*MMACHC*	Methylmalonic aciduria complicated with hyper cystinuria	609831	*MMACHC*	NM_015506.3	10 (2.2)
*PAH*	Phenylketonuria (PKU)	612349	*PAH*	NM_000277.3	8 (1.8)
*GALC*	Krabbe disease	606890	*GALC*	NM_000153.4	8 (1.8)
*MLC1*	Macrocephalic leukoencephalopathy type 1 with subcortical cyst (MLC1)	605908	*MLC1*	NM_015166.4	6 (1.3)
*UNC13D*	Familial hemophagocytic lymphohistiocytosis	608897	*UNC13D*	NM_199242.3	5 (1.1)
*CAPN3*	Limb muscular atrophy	114240	*CAPN3*	NM_000070.3	5 (1.1)
*PKHD1*	Autosomal recessive polycystic kidney (ARPKD)	606702	*PKHD1*	NM_138694.4	5 (1.1)

^a^ The genomic reference sequence used is GRCh37 (Genome Reference Consortium human genome assembly 37).

## 4 FRAGILE X SYNDROME— *FMR1* GENE DETECTION RESULTS

A total of 488 female patients underwent FMR1 gene detection, and there were a total of 6 patients with FMR1 gene mutation (1.2%) (6/488). There were 4 patients (2 patients with *n* = 45, 1 patient with *n* = 53, and 1 patient with *n* = 54) with FMR1 gray region mutation, accounting for 0.8% (4/488), 1 patient (*n* = 105) with former mutation, accounting for 0.2% (1/488), and 1 patient (*n* > 200) with all mutations, accounting for 0.2% (1/488). The mutations in the FMR1 gene are shown in Table 2.

**TABLE 2:** The classification of the FMR1 gene test results.

FMR1 gene type	CGG number of repetitions (*n*)	Number of cases (*n*)	Frequency (*n*)
Normal type	<45	482	98.77
Intermediate type	45–54	4	0.82
Premutation	55–200	1	0.20
Full mutation	>200	1	0.20

## 5 SPINAL MUSCULAR ATROPHY— *SMN1* GENE DETECTION RESULTS

**FIGURE 2:**
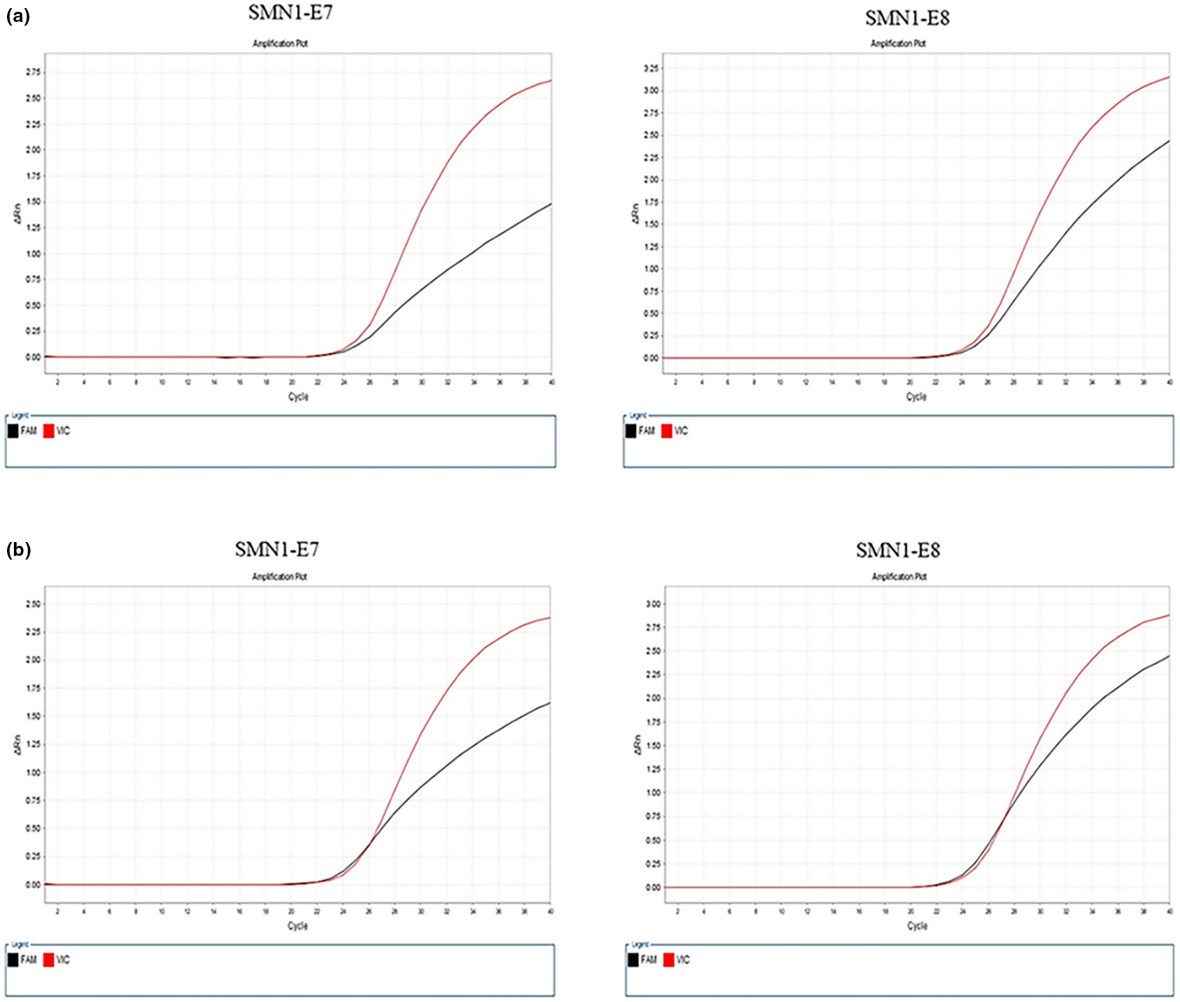
(a) Carrier Detection of *SMN*1‐E7 and *SMN*1‐E8. (b) Patient with normal genotype Detection of *SMN*1‐E7 and *SMN*1‐E8.

Among the 415 women screened for spinal muscular atrophy—*SMN1* gene carrier, 13 patients (3.1%) with *SMN1* gene carrier were detected, including 11 males and 2 carriers, i.e., two parents carried the SMN1 pathogenic mutation gene concurrently, and the detection rate was 0.5%. The gene test results of the carriers and patients with normal genotype are shown in Figure 2a,b, Tables 3 and 4.

**TABLE 3:** The Exon‐7 of SMN1.

Sample	CtFAM	CtVIC	ΔCT	RQ
Carrier 1	24.3	24.0	0.7	0.6
Carrier 2	24.6	24.2	0.8	0.6
Carrier 3	24.8	24.4	0.8	0.6
Genotype normal	24.1	24.4	0.02	0.9

*Note*: (1) RQ = 2^−ΔCT^; (2) No deletion of SMN1 exon 7 was detected: RQ ≥ 0.8; critical value: 0.65 < RQ < 0.8; SMN1 exon 7 deletion heterozygous: 0.45 < RQ ≤ 0.65; SMN1 exon 7 deletion homozygous: RQ < 0.25 or FAM has no signal.

**TABLE 4:** The Exon‐8 of SMN1.

Sample	CtFAM	CtVIC	ΔCT	RQ
Carrier 1	24.3	24.5	0.8	0.6
Carrier 2	23.9	24.1	0.9	0.6
Carrier 3	24.6	24.2	0.9	0.5
Genotype normal	23.8	24.2	0.06	0.9

*Note*: RQ = 2^−ΔCT^.

In this study, the spouses of 11 female carriers were screened for the *SMN1* gene, with an acceptance rate of 84.6%. The spouses of two female carriers disagreed with the carrier screening because (1) they were willing to take the risk with the hope of being lucky, as the probability that the spouses were both carriers of the *SMN1* mutation gene was low; (2) they were worried that if they found out the carrier status, they would face discrimination from their relatives and the mental burden will also increase; (3) after explanation and consultation, they still retained doubts about the examination results.

## 6 CLINICAL OUTCOMES OF HIGH‐RISK GROUPS BY DETECTION

In this study, we detected that 1 male partner and female partner pair carried the AGL (glycogen storage disease type III) mutation gene, and 2 pairs were both carriers of the SMA pathogenic gene, 1 patient was a carrier of a full mutation of the FMR1 gene, 1 patient carried the pre‐FMR1 mutation, and 4 patients were carriers of the FMR1 intermediate type. We provided these patients with genetic counseling for good prenatal and postnatal care and selection of a pregnancy assistance protocol. Among them, parents with *AGL* carriers requested for prenatal diagnosis in the second trimester, and the results indicated that twin pregnancies had normal genotypes and no abnormalities were found in the follow‐up. A parent with *SMA* carriers chose PGT‐M to assist pregnancy, and made prenatal diagnosis in the second trimester, and gave birth to a female offspring with normal genotype. Another parent with *SMA* carriers finally gave up pregnancy because they were worried about the risks of their child being a carrier and suffering from the disease. Two patients with *FMR1* intermediate type suffered from fragile X‐related POI. After IVF‐ET assisted pregnancy, they all gave birth to offspring with normal genotype, and no abnormality was found during the follow‐up. The patients with *FMR1* pre‐mutation were diagnosed in the second trimester after IVF‐ET assisted pregnancy, and a male offspring with normal *FMR1* genotype was delivered. There was no abnormality in the follow‐up. The female patients with complete mutation of *FMR1* gene refused PGT‐M due to financial reasons and underwent prenatal diagnosis in the second trimester after resuscitation and transplantation of an embryo. The results showed that the *FMR1* genotype of the fetus was normal, and a baby boy was delivered at full term. The re‐examination of peripheral blood showed that the *FMR1* genotype was normal. In addition, we performed *FMR1* gene screening on the immediate family members of a woman with an all‐mutated *FMR1* gene, and the *FMR1* genotype of her father, brother, and daughter were all normal.

## 7 DISCUSSION

The purpose of carrier screening is to determine whether individuals with normal phenotypes have a pathogenic gene for an autosomal or X‐linked recessive hereditary disease. Usually individual carriers are not affected by the pathogenic genes or have clinical symptoms, but they are at risk of having an offspring with the disease. If both the male partner and female partner are carriers of the same autosomal recessive hereditary disease (i.e., carrier parent), the probability of having a child with a hereditary disease is 25% and the probability of having their offspring as a carrier is 50%. For X‐linked diseases, 50% of male offspring born to female carriers develop the disease, and 50% of the female offspring are carriers. Single‐gene disease carrier screening has become an effective method to identify high‐risk parents, the critical role such screening plays in the provision of meticulous reproductive counseling, thereby promoting informed reproductive autonomy. Doing so endows affected parents with a spectrum of reproductive alternatives, enabling them to make enlightened decisions concerning their reproductive journey, and can be used as a primary prevention measure to prevent birth defects. At present, there is insufficient awareness and education on pre‐pregnancy carrier screening in China, and the experience and facilities for carrying out the project are not perfect enough. There are no clear standards and guidelines to guide clinical application. Therefore, to provide more clear reproductive choices for high‐risk parents, we should implement the research plan to expand pre‐pregnancy carrier screening and formulate a screening model suitable for the Chinese population.

In 2015, Igenomix, the largest assisted reproductive center in Europe, conducted pre‐pregnancy carrier screening on 2570 participants. The results of the screening revealed a total of 623 participants carrying genes of recessive and X‐linked hereditary diseases, and 5% (7/138) of the parents who were ready to receive assisted reproductive pregnancy were found to carry the same pathogenic gene mutation (Martin et al., 2015). In 2020, Xi et al. performed carrier screening of 201 genes (121 autosomal recessive diseases and 14 X‐linked diseases) on 1462 parents who underwent assisted reproductive pregnancy (Xi et al., 2020). Among the parents, 2.26% were identified as high‐risk parents. This study included 129 genes that can be screened and infertile patients and patients receiving ART assisted pregnancy were screened for being carriers. The results indicated that the detection rate of high‐risk parents carrying the same pathogenic gene was 0.3%, which was lower than that reported outside of China. This may be related to regional differences, ethnicity, target population, sample size, and types of diseases included. In this study, the male partner was screened only when the female partner was found to possess the pathogenic gene; hence, the number of men who underwent genetic testing for 110 kinds of genes was small, but all these studies showed that the promotion of ECS in the population was of great clinical significance. In real‐life scenarios, there are instances where certain parents may express a desire to acquire such knowledge but consciously decide not to pursue any course of action. They may choose to engage in a level of “risk‐taking” or even demonstrate preparedness to accept the potential occurrence of offspring with genetic disorders. Consequently, it should be noted that undergoing genetic testing does not invariably result in subsequent behavioral modifications.

In this study, the detection rate of at least 1 pathogenic gene mutation in the overall research population who underwent the expanded 108 monogenic tests was 31.7% (145/458). It was higher than the detection rates of Franasiak et al. (2016), Lazarin et al. (2013), and Zhao et al. (2021) (31.7% vs. 25.10%, 24.0% and 18.23%), but lower than the detection rates of Guo and Gregg (2019). and Xi et al. (2020) (31.7% vs. 32.6%–62.9% and 46.73%). The carrier rate of the *SMN*1 gene in 426 women of childbearing age was 3.5%, higher than that in Liuzhou City, Guangxi Province (1.2%) (Tan et al., 2018), Foshan City, Guangdong Province (1.48%) (Zhou et al., 2022), Taiwan Province, China (2.2%) (Su et al., 2011), and western countries (2.5%) (Lyahyai et al., 2012). This may be related to the sample size, screening pattern, use of different carrier screening panels and platforms, and so on, in addition to different regions and ethnicities. Furthermore, when analyzing the carrier status of different pathogenic genes, the results for Krabbe's disease (*GALC*), autosomal recessive polycystic kidney (*PKHD*1), and leukoencephalopathy (*MLC*1) are similar to those reported by Zhao et al. (2021), and there was a high detection rate.

In 2017, the ACOG Committee proposed criteria for the inclusion of disease types in Expanded Carrier Screening (ECS) (Committee on Genetics, 2017): (1) carrier frequency of 1/100 or higher, clear phenotype with adverse impact on quality of life, leading to pregnancy or physical harm, necessitating surgical or medical intervention, or early‐onset diseases; (2) for individuals with a family history of Fragile X‐related disorders or intellectual disability suggestive of Fragile X syndrome, or females with a personal history of ovarian dysfunction, screening for Fragile X syndrome‐FMR1 gene mutations is recommended; (3) carrier screening should include screening for spinal muscular atrophy. Diseases suitable for carrier screening may encompass the following (Henneman et al., 2017): (1) autosomal recessive (AR) disorders: diseases causing severe congenital malformations and disabilities. (2) Some ECS also includes X‐linked diseases, such as Fragile X syndrome and Duchenne muscular dystrophy (DMD). Consequently, this study selected 110 monogenic diseases for preconception carrier screening projects, based on each gene or related disease possessing the following characteristics: (1) severe clinical symptoms; (2) high carrier rates of pathogenic genes in the screening population; (3) reliable testing methods with high specificity and sensitivity; (4) genetic counseling and prenatal diagnosis can be conducted upon detection of abnormalities in these genes, preventing the birth of affected children.

Relevant literature have reported that the incidence of *FMR*1 gene mutation varies greatly due to different regions and populations, and there are also differences among ethnic groups (Liu & Fragile, 2017; Sha et al., 2016; Ye et al., 2014). The results of this study showed that among 488 women of childbearing age, there were 4 patients with *FMR*1 intermediate type, and the detection rate was 1:122, which was lower than 1:98 in Urumqi reported by Liu and Fragile (2017). and higher than 1:137 in South Korea (Jang et al., 2014) and 1:345 in Shenzhen (Chen et al., 2019). There was 1 case of pre‐mutation, and the detection rate was 1:488, which was higher than that in South Korea and Shenzhen. On the one hand, these results may be due to geographical, ethnic, and sample size differences; on the other hand, they may also be because the cohort targeted in this study was infertility patients, with some having adverse pregnancy and childbirth history, and some having ovarian hypofunction. This also fully illustrates that *FMR*1 gene mutation accounts for a relatively high proportion in the infertility population. Therefore, among infertile people, especially patients receiving assisted reproductive technology (ART) for pregnancy, to provide prospective parents with the knowledge and options necessary to make informed and autonomous decisions regarding their reproductive choices, it is necessary to expand the screening range of the *FMR1* gene.

In this study, genetic counseling and fertility guidance were provided to the identified high‐risk individuals or parents, thus reducing the risk of parents with the *SMN1* carriers passing the disease‐causing gene to their offspring. For patients who visited the reproductive center, “history collection‐routine examination‐pre‐pregnancy gene screening” was performed, pre‐pregnancy gene screening was included in the routine examination before ART treatment, early genetic counseling was provided to the high‐risk parents, and they were provided a myriad of responsible and ethically sound reproductive choices. Carrier screening, especially pre‐pregnancy carrier screening, can play a major role in fostering informed choices and enabling enhanced quality of life in the field of human assisted reproduction.

In summary, in this study, we preliminarily identified the diseases with high carrier rate in the region through the screening of pre‐pregnancy‐related genes among women of childbearing age. The results of this study provide a reference for the design of a panel for carrier screening of people in this region and a basis for future genetic counseling. At the same time, early intervention, PGT‐M, or prenatal diagnosis should be carried out for high‐risk parents; it will provide families at risk of having a child with a genetic disorder informed and more reproductive options.

## AUTHOR CONTRIBUTIONS

Li Tan, Yuefan Qi and Peijuan Zhao conceived the idea and conceptualized the study. Li Tan, Peijuan Zhao, Yuefan Qi, Guo Yu, Dongmei Zhao, Yun Gai Xiang and Yu Xia Song collected the data. Li Tan, Peijuan Zhao, Yuefan Qi, LanLan Cheng, Yun Gai Xiang, Dongmei Zhao, Yu Xia Song and Guo Yu analyzed the data. Li Tan and Peijuan Zhao drafted the manuscript, then Li Tan and Peijuan Zhao reviewed the manuscript. All authors read and approved the final draft.

## FUNDING INFORMATION

Wu Jieping Medical Foundation Special Fund for Clinical Research [320.6750.18558(9)].

## CONFLICT OF INTEREST STATEMENT

The authors declare that they have no competing interests.

## ETHICS APPROVAL AND CONSENT TO PARTICIPATE

I confirm that I have read the Editorial Policy pages. This study was conducted with approval from the Ethics Committee of The Second Affiliated Hospital of Zhengzhou University (Approval number: 2022373). This study was conducted in accordance with the declaration of Helsinki. Written informed consent was obtained from all participants.

## Data Availability

The data used to support the findings of this study are available from the corresponding author upon request.
